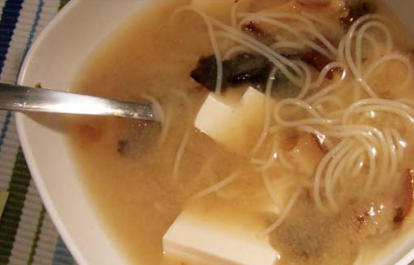# Diet and Nutrition: A Spoonful of Risk?

**Published:** 2007-07

**Authors:** Valerie J. Brown

Soy foods are rich in isoflavones such as genistein and daidzein, strong antioxidants that bind to cells’ estrogen receptors. For years, soy products have presented intriguing hints that they may confer protection against some cancers. For example, the incidence of prostate cancer among Asian males is far below that of U.S. males, with the former eating far more soy products than the latter. Now a large study offers more intriguing—and more confusing—evidence than ever.

Norie Kurahashi and colleagues at Japan’s National Cancer Center used data from a 1995 cohort of the Japan Public Health Center-Based Prospective Study on Cancer and Cardiovascular Diseases to analyze diet and prostate cancer data for 43,509 Japanese men. Participants aged 45–74 had answered questions on medical history, lifestyle factors such as smoking, and how often they ate each of 147 foods. At the end of 2004, there were 307 recently diagnosed cases of prostate cancer in the cohort, of which 74 were advanced, 220 were localized to the prostate gland, and 13 were of undetermined stage.

The Kurahashi group found a dose-dependent lowering of risk of localized prostate cancer with increasing consumption of soy foods. However, further analysis showed that consumption of miso soup was associated with increased risk of advanced prostate cancer—men over age 60 who ate two or more bowls of miso soup a day were at twice the risk of advanced cancer as those who ate less than one bowl. And for men under 60, the risk for both localized and advanced prostate cancer rose with consumption of genistein, daidzein, and soy foods in general. The findings were published in the March 2007 *Cancer Epidemiology, Biomarkers and Prevention*.

Richard Hoffman, an associate professor of medicine at the University of New Mexico, points out that the Kurahashi study did not adjust for family history, a known risk factor for prostate cancer. Men with a family history of prostate cancer might try to prevent cancer by increasing their isoflavone intake. Therefore, he says, family history could confound the study results, producing a spurious association between higher isoflavone consumption and increased risk of advanced cancer.

Another possible confounder is that isoflavones, which are weakly estrogenic, inhibit testosterone production, thus limiting tumor growth but only as long as cells have estrogen receptors. However, as tumor cells develop, they lose those receptors, freeing up testosterone and making the tumors more aggressive.

Regarding the apparent protective effect against localized cancer, Kurahashi says, “Isoflavones might delay the progression from latent to clinical[ly] significant prostate cancer in Japanese [men]. However, when or how isoflavones affect latent or localized prostate cancer development and whether isoflavones can be used in the treatment or chemoprevention of this cancer are not yet clear.” Kurahashi and colleagues will follow the cohort through two full decades and conduct nested case–control studies using archived blood samples in hopes of solving this puzzle.

## Figures and Tables

**Figure:**